# The Impact of Virtual Consultations on Quality of Care for Patients With Type 2 Diabetes: A Systematic Review and Meta-Analysis

**DOI:** 10.1177/19322968251316585

**Published:** 2025-02-17

**Authors:** Reham Aldakhil, Geva Greenfield, Elena Lammila-Escalera, Liliana Laranjo, Benedict W. J. Hayhoe, Azeem Majeed, Ana Luísa Neves

**Affiliations:** 1 Department of Primary Care and Public Health, Imperial College London, London, UK; 2 Westmead Applied Research Centre, Faculty of Medicine and Health, The University of Sydney, Sydney, NSW, Australia

**Keywords:** equity in health care, meta-analysis, patient satisfaction, telemedicine, type 2 diabetes virtual consultations

## Abstract

**Background:**

Virtual consultations (VC) have transformed healthcare delivery, offering a convenient and effective way to manage chronic conditions such as Type 2 Diabetes (T2D). This systematic review and meta-analysis evaluated the impact of VC on the quality of care provided to patients with T2D, mapping it across the six domains of the US National Academy of Medicine (NAM) quality-of-care framework (ie, effectiveness, efficiency, patient-centeredness, timeliness, safety, and equity).

**Methods:**

A systematic search was conducted in PubMed/MEDLINE, Cochrane, Embase, CINAHL, and Web of Science for the period between January 2010 and December 2024. Eligible studies involved adult T2D patients, evaluated synchronous VCs, and reported outcomes relevant to NAM quality domains. Two independent reviewers performed screening, and studies were assessed using the Mixed Methods Appraisal Tool (MMAT). A narrative synthesis was conducted for each quality domain, and a meta-analysis of HbA1c levels was performed using random-effects models.

**Results:**

In total, 15 studies involving 821 014 participants were included. VCs were comparable with face-to-face care in effectiveness, efficiency, patient-centeredness, and timeliness, with improvements in accessibility and patient satisfaction. Mixed results were found for safety due to limitations in physical assessments, and for equity, with older adults and those with lower digital literacy facing more challenges. The meta-analysis showed no significant difference in HbA1c reduction between VCs and face-to-face (standardized mean difference [SMD] = -0.31, 95% confidence interval [CI]: -0.71 to 0.09, *P* = 0.12).

**Conclusion:**

VCs offer a promising alternative to in-person care, but addressing digital disparities and improving access for older adults are essential for maximizing VC potential.

## Introduction

Around 463 million individuals worldwide have diabetes, representing 9.3% of people aged between 20 and 79 years. Projections suggest an increase to 578 million (10.2%) by 2030 and to 700 million (10.9%) by 2045.^1,2^ Type 2 diabetes (T2D) constitutes over 90% of those diagnosed with diabetes.^3^ T2D is linked to the development of both microvascular and macrovascular complications, leading to significant psychological and physical morbidity for both patients and their caregivers.^4^ T2D also imposes a substantial burden on health care systems including increased hospital stays, higher medication costs, and the need for imputations and other cardiovascular surgical interventions.^5,6^ Regular monitoring in primary care and specialist consultations are necessary for effective management, adding to health care expenses.^7^

Traditionally, healthcare providers in primary and secondary care settings have monitored their patients with T2D primarily through in-person consultations. The COVID-19 pandemic has hastened the digital transformation in health care,^8-10^ with virtual consultations (VC) gaining significant attention as an alternative service delivery model.^11,12^ Postpandemic, this model of care continues, especially in the management of chronic conditions such as T2D.^13,14^ These consultations can be facilitated synchronously, in real-time, through videoconferencing or phone calls, or asynchronously, where patients upload their health information to a digital platform, with practitioners subsequently providing feedback or therapeutic recommendations at their convenience.^15^ In this review the VC are defined as synchronous, real-time interactions between health care providers and patients using phone or video technology, enabling remote clinical assessment, management decisions, and patient education in a nature that is sufficient to meet the key components of the consultation when conducted via face-to-face interaction, this aligned with the definition provided by the American Medical Association (AMA).^16^

Virtual consultations can enhance appointment attendance and potentially decrease health care costs for T2D patients.^17-19^ While VC, particularly when synchronous, have also reported a reduction in HbA1c levels, there remains considerable variability in these findings.^20,21^ While the empirical evidence supports the favourable outcomes and benefits of VC, they also have limitations.^22,23^ Concerns about virtual care include low digital literacy among older patients^24-26^ and potential limitations in tailoring treatments, carrying out comprehensive assessments, and communication barriers.^8,27^ In addition, VC introduces data privacy and security concerns.^8,28,29^ While the adoption of VC has generally increased, uptake remains lower among older people, ethnic minorities, and individuals from lower socioeconomic backgrounds,^30^ indicating inequities between patient groups.^31^

With the increasing use of VC continuing across a range of health sectors, it is crucial to understand its impact on the quality of care in common long-term conditions such as T2D. While VC show promise, significant gaps exist in understanding their impact on comprehensive quality of care, particularly regarding long-term clinical outcomes, safety and risk management, resource utilization, and equity implications. A comprehensive analysis is essential to guide health care policies and regulatory frameworks related to VC, to shape best practice guidelines,^32,33^ and to further understand the implications on health care delivery, patient engagement, and cost-efficiency.^34^

The aim of this review is to systematically summarize the evidence on the impact of VC on the quality of care for T2D patients, mapping impacts against the six domains of quality proposed by the US National Academy of Medicine (NAM) quality framework (ie, effectiveness, efficiency, patient-centeredness, timeliness, safety, and equity).

## Methods

### Search Strategy

A systematic literature search was conducted to identify studies that examined the impact of VC on the quality of care for adult patients with T2D. The databases searched included PubMed/MEDLINE, Cochrane, Embase, CINAHL, and Web of Science, and covered studies published between January 2010, and December 2024, this period was selected as it captured the period of digital healthcare technology maturity, and global adoption of virtual care, driven by advancements in telemedicine infrastructure and integration into health care systems.^9,12^ A comprehensive search strategy was constructed, structured around three key concepts: virtual care, T2D and the NAM’s six domains of care quality (ie, patient-centeredness, efficiency, safety, effectiveness, timeliness, and equity). Although the review protocol allowed for the inclusion of non-English studies, no non-English studies were ultimately included in the review, however, non-English full-texts were to be reviewed by bilingual team members or an external professional translation service. The search included both peer-reviewed and grey literature. Grey literature was identified through Google Scholar and institutional repositories (eg, National Institutes of Health [NIH] RePort, and OpenGrey.eu) using the same search criteria. Key search terms used included combinations of the concepts telemedicine, telehealth, virtual consultation, T2D, and each of the quality domains; detailed search strings for each database are provided in Appendix A. The detailed study protocol is registered with PROSPERO (Registration number: CRD42023474219), and has been previously published in BMJ Open.^35^

### Eligibility Criteria

Studies focusing on T2D adult patients that involved synchronous consultations were included. The outcomes evaluated had to include at least one of the NAM’s quality-of-care domains. Cross-sectional studies were included if they reported outcomes of interest relevant to the quality domains, such as patient satisfaction or health care access. A detailed description of the inclusion and exclusion criteria is provided in Table 1.

**Table 1.** Inclusion and Exclusion Criteria.

	Inclusion	Exclusion
***Population***	Adults diagnosed with T2D.	Studies involving with type 1 or gestational diabetes unless they provided a subgroup analysis for type 2 diabetes.
***Intervention***	Real-time (synchronous) virtual consultations via telephone or videoconference, including studies where the interventions were part of multicomponent strategies but with clear outcomes of virtual consultation.	Studies focus solely on asynchronous telehealth interventions like text messaging, email consultation, or live chat.
***Comparator***	In-person, face-to-face consultations.	No face-to-face comparison group or indication of a comparison
***Outcomes***	Measures related to the NAM quality domains such as patient satisfaction, efficiency, effectiveness, and safety.	Studies reporting only qualitative outcomes or those not aligning with the NAM domains.
***Study Designs***	Randomized controlled trials, cluster-randomized trials, quasi-experimental studies, case-control studies, cohort studies, cross-sectional and cost effectiveness studies.	Editorials, commentaries, case reports, and reviews

The table summarizes the criteria used to include or exclude studies, specifying the required characteristics for inclusion and the conditions for exclusion.

Abbreviations: T2D, Type 2 Diabetes; NAM, National Academy of Medicine.

### Study Selection

Title, abstract and full-text review screening were performed using the software Covidence (Covidence, Melbourne). Two reviewers (RA and EL) independently screened the titles and abstracts of studies identified during the initial search based on the inclusion and exclusion criteria. Any discrepancies between reviewers were resolved through discussion. Cohen’s Kappa was calculated to assess the interrater reliability between the two reviewers at two stages: the title and abstract stage, and the full text screening stage. A Kappa value of ≤0 indicates no agreement, 0.01 to 0.20 as slight, 0.21 to 0.40 as fair, 0.41 to 0.60 as moderate, 0.61 to 0.80 as substantial, and 0.81 to 1.00 as almost perfect agreement.^36^

### Data Extraction

A standardized data extraction form was used to collect relevant data from the included studies, including authors, publication year, country, study design, sample size, participants, type of consultation, consultation description, domain(s) of quality care, outcome measures relevant to NAM domains, and main findings.

### Quality Assessment

The quality of the included studies was assessed using the Mixed Methods Appraisal Tool (MMAT) version 18^37^ (Appendix B). Two independent reviewers (RA and EL) performed the assessment, and disagreements were resolved by consensus. Scores were assigned as follows: “Yes” = 1, “No” = 0, and “Can’t tell” = 0.5. A study was considered as high risk if its average score was < 0.5, moderate risk if between 0.5 and 0.8, and low risk for scores of 0.8 or higher.

### Synthesis of Results and Meta-Analysis

A narrative synthesis of all selected studies was performed, mapping the outcomes of the studies to each NAM domain and summarizing the relevant outcome data into summary tables. Continuous outcomes evaluated in eight of the 15 included studies (ie, glycated hemoglobin; HbA1c) were pooled using random-effects models. The random-effects model accounted for heterogeneity among the included studies. Sensitivity analyses were conducted based on the risk of bias, categorizing studies into different risk levels (ie, low, moderate, high). The presence of publication bias was assessed by a funnel plot.

## Results

### Search and Screening Results

**Figure 1.**
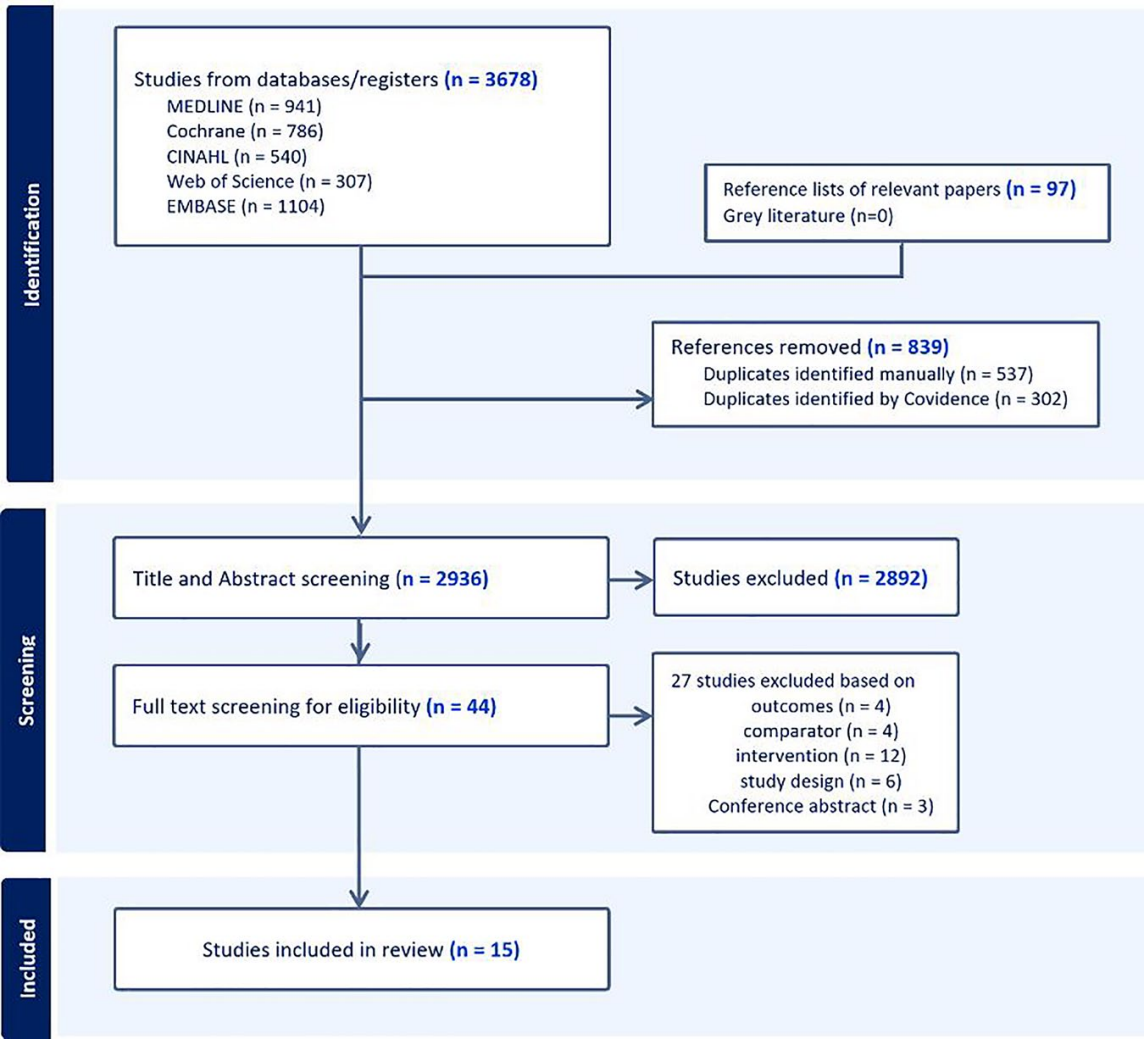
PRISMA (Preferred Reporting Items for Systematic Reviews and Meta-Analyses) flowchart. Flow diagram summarizing the identification, screening, and inclusion process for studies in the systematic review. It outlines the number of studies identified, screened, excluded, and included in the final analysis.

The initial search retrieved a total of 3678 records. No relevant records were found from searching grey literature, and 97 papers were added from the reference lists of relevant articles, giving 2936 papers for screening. For the title and abstract screening, Cohen’s Kappa was calculated as 0.92, indicating almost perfect agreement between the reviewers. During the full text screening, Cohen’s Kappa was 0.79, reflecting substantial agreement. Following full-text screening, 15 papers met the inclusion criteria Figure 1.

### Characteristics of the Included Studies

The 15 included studies included a total of 821 014 participants. The sample size ranged between 100 and 733 006 participants, with publication years ranging from 2013 to 2023. Study designs included retrospective cohort studies (n = 8),^38-45^ randomized controlled trials (n = 3),^46-48^ cross-sectional (n = 2),^49,50^ quasi-experimental (n = 1),^51^ and prospective single-cohort study (n = 1).^52^ Eight studies were conducted in the United States,^38,40-42,45,48,50,51^ two in Saudi Arabia,^39,52^ and one each in Canada,^49^ Denmark,^47^ Singapore,^44^ Australia,^46^ and Italy.^43^

The modalities of the consultations across the 15 included studies varied, with 40% (n = 6) offering a mix of telephone and video.^38,41-43,45,49^ Video calls were also used in 40% (n = 6) of the studies.^40,41,46^-^48,50^ Telephone-only consultations represented 20% (n = 3) of the studies (Table 2).^39,44,52^

**Table 2.** Characteristics of the Included Studies.

Author, year	Country	Study design	Study duration	Age	Ethnicity	Gender	Sample size	Participants	Consultation type	Consultation description	Domain(s) of quality care
*Adhikari et al, 2023*	United States	Retrospective Cohort Study	Three years	Mean 67 years (SD = 12)	White 69.0% Black 21.0% Hispanic 6.6% Asian 0.9% Native Hawaiian or other Pacific 1.1% American Indian or Alaska Native 1.0% Missing 5.9%	94.0% Male 6.0% Female	733006	Veterans with recently diagnosed diabetes	Telephone or Video	Categorized according to decision support identifier; no further details provided	Equity
*Amy et al, 2021*	United States	Retrospective Cohort Study	Not Specified	Mean 68.3 years	White: 77%, Black: 12%, Hispanic: 3%, Other/Unknown: 10%	96% Male	9010	Patients with type 2 diabetes at VA primary care clinics	In-clinic Video Calls	Remote primary care providers assigned a patient appointment via in-clinic synchronous videoconferencing appointment with a high-definition camera and digital stethoscope operated by a trained nurse; supplemented by quarterly in-person visits	Effectiveness, Patient-Centeredness
*AlMutairi et al, 2021*	Saudi Arabia	Retrospective Chart Review	Three months	Between 50 and 70 years (58%)	Not reported	48.5% Male 51.5% Female	200	Adult patients with uncontrolled T2D (HbA1c > 9)	Telephone Calls	Remote management by the same clinical team as in-person care, including reviewing therapeutic plans, patient counseling	Effectiveness, Efficiency
*Baker et al, 2019*	United States	Retrospective Cohort Study	18 months	FTF: 65.6 ± 6.1 years Video: 63.3 ± 6.6 years	FTF: White 82% African American 15% Other 3% Video: White 86% African American 14% Other 0%	FTF: 99% Male 1% Female Video: 100% Male	103	Patients with T2D and HbA1c>7%	Video Calls	Pharmacist reviews patient’s medical records in real-time, discusses health status, medication adherence, and new symptoms; demonstrates medication techniques and discusses treatment plans	Effectiveness, Patient-Centeredness
*Beamish et al, 2023*	Canada	Cross-sectional Survey	13 months	Mean 60.4 years (SD = 14.5)	Caucasian 87% Middle Eastern 3% Black 2.4% Southern Asian 2.1% Chinese 1.5% Other 6.3%	54% Male, 46% Female	333	Adult patients with diabetes care	Telephone and Video Calls	Virtual care encounters are provided primarily over the phone by the multidisciplinary clinic	Efficiency, Safety, Patient-Centeredness
*Fatehi et al, 2015*	Australia	Randomized Control Study	10 months	Mean 57 years (SD = 14)	Not reported	66% male, 34% female	73	English speaking adults with diagnosed type 1 or 2 diabetes	Video Calls	Two consultations by different endocrinologists during the same clinic session; in the telemedicine group, consultations were assigned by randomization	Effectiveness
*Herber E. et al, 2023*	United States	Retrospective Cohort Study	Seven months	FTF: 60.5 ± 11.5 years Hybrid: 60.9 ± 11.3 years Telehealth: 61.4 ± 12.3 years	White 65% Black 27% Other 8% across all groups	Female: FTF: 46.1% Hybrid: 48.6% Telehealth: 54.6%	1237	Patients with HbA1c > 8% and at least 18 years old	Telephone or Video	No specific details on how the consultation was conducted	Effectiveness
*Koh ZWJ et al, 2023*	Singapore	Retrospective Cohort Study	Five months	FTF: Mean 60.87 ± 9.86 years Telephone: Mean 60.61 ± 8.79 years	Chinese 79.0% Malay 9.6% Indian 9.9% Others 1.4%	FTF: 49.7% Male 50.3% Female Telephone: 52.8% Male 47.2% Female	644	Patients with type 2 diabetes and HbA1c ≥ 7.0%, aged 21-80 years	Telephone Consultations	Index consultation could be either face-to-face or telephone, defined by the first consultation during the study period	Effectiveness
*Rasmussen et al, 2015*	Denmark	Randomized Control Study	Nine months	64.6 years (range 36-80)	Not reported	FTF: 35% Male 20 % Female Video: 32.5% Male 12.5% Female	40	T2D patients visiting the outpatient clinic of the Endocrinology Department	Video Calls	Treatment at home by video conferences only, using a broadband solution installed by the Danish Telephone Company	Effectiveness
*Robinson et al, 2016*	United States	Cross-sectional Survey	Three months intervention, three-month follow-up	Mean 52.1 years	White 78% African American 22% Latin American 11%	55% Male 45% Female	34	Patients ≥18 years old with uncontrolled T2D (HbA1c level of ≥9%)	Video Calls	Virtual visits with a nutritionist, pharmacist, and clinical psychologist using FaceTime on an iPad2; initial visits lasted about one hour, with follow-ups around 30 minutes	Patient-Centeredness
*Russo et al, 2022*	Italy	Retrospective Cohort study	12 months	FTF: 69.1 ± 11.1 years Telehealth: 71.3 ± 11.2 years	Not reported	FTF: 58.6% Male 41.4% Female Telehealth: 54.1% Male 45.9% Female	411322	Patients with T2D	Not Reported	Telemedicine consultations identified by specific annotations in the patient’s electronic chart	Effectiveness, Safety
*Shao et al, 2023*	United States	Quasi-experimental Difference-in-Difference Study	24 months	Mean 64.5 years	White 48.8% Black 45.7% Hispanic 3.4% Other 2.1%	41.3% Male 58.7% Female	28578	Adults aged 35 years and older with type 2 diabetes	Telephone or Video	Telehealth visits identified by the encounter type in the Common Data Model, including virtual visits conducted via video, telephone, or other means	Effectiveness
*Shea et al, 2013*	United States	Randomized Control Study	6+ years	FTF: Mean 70.9 years Video: 70.8 years	White FTF: 50.6%, Video 48.2% Hispanic FTF: 34.6%, Video 35.8% African American FTF: 14.5%, Video: 15.3% Other FTF: 0.2%, Video: 0.7%	37% Male 63% Female	1665	Patients aged 55 years or older with diabetes residing in medically underserved areas	Video Calls	Home telemedicine unit for videoconferencing with nurse case managers, glucose and blood pressure monitoring	Effectiveness, Equity
*Tourkmani et al, 2023*	Saudi Arabia	Prospective Single-Cohort	Four months	Mean 57 ± 12 years	Not reported	Not reported	130	Patients with T2D having an HbA1c value >9	Phone Calls	Phone calls for communication; WhatsApp used for providing written instructions, educational materials, and audio-visual aids; SMS for SMBG readings	Effectiveness
*Ward et al, 2023*	United States	Retrospective Cohort Study	30 months	Mean 59.4 years (SD = 11.65)	Black 73.23% White 24.99% Other 1.78%	39.82% Male 60.2% Female	2319	Patients with T2D from January 2019 to June 2021	Telephone or Video	Synchronous consultations with a health care professional using real-time, audio/video communication	Effectiveness

This table details the key features of the included studies, including study design, sample size, intervention type, patient population, follow-up duration, and primary outcomes.

Abbreviations: HbA1c, Hemoglobin A1c; NAM, National Academy of Medicine; SMBG, Self-Monitoring of Blood Glucose; T2D, Type 2 Diabetes; VA, Veterans Affairs; FTF, Face to Face.

### Risk of Bias Assessment

Of the 15 studies, nine (60%) had a moderate risk of bias,^38,39,42-44,50,51,53^ four (27%) had a low risk,^41,46,49,48^ and two (13%) had a high risk of bias.^47,52^ Appendix C shows the risk of bias assessments for nonrandomized, randomized controlled trials, quantitative, and mixed methods studies.

### Effectiveness

**Figure 2.**
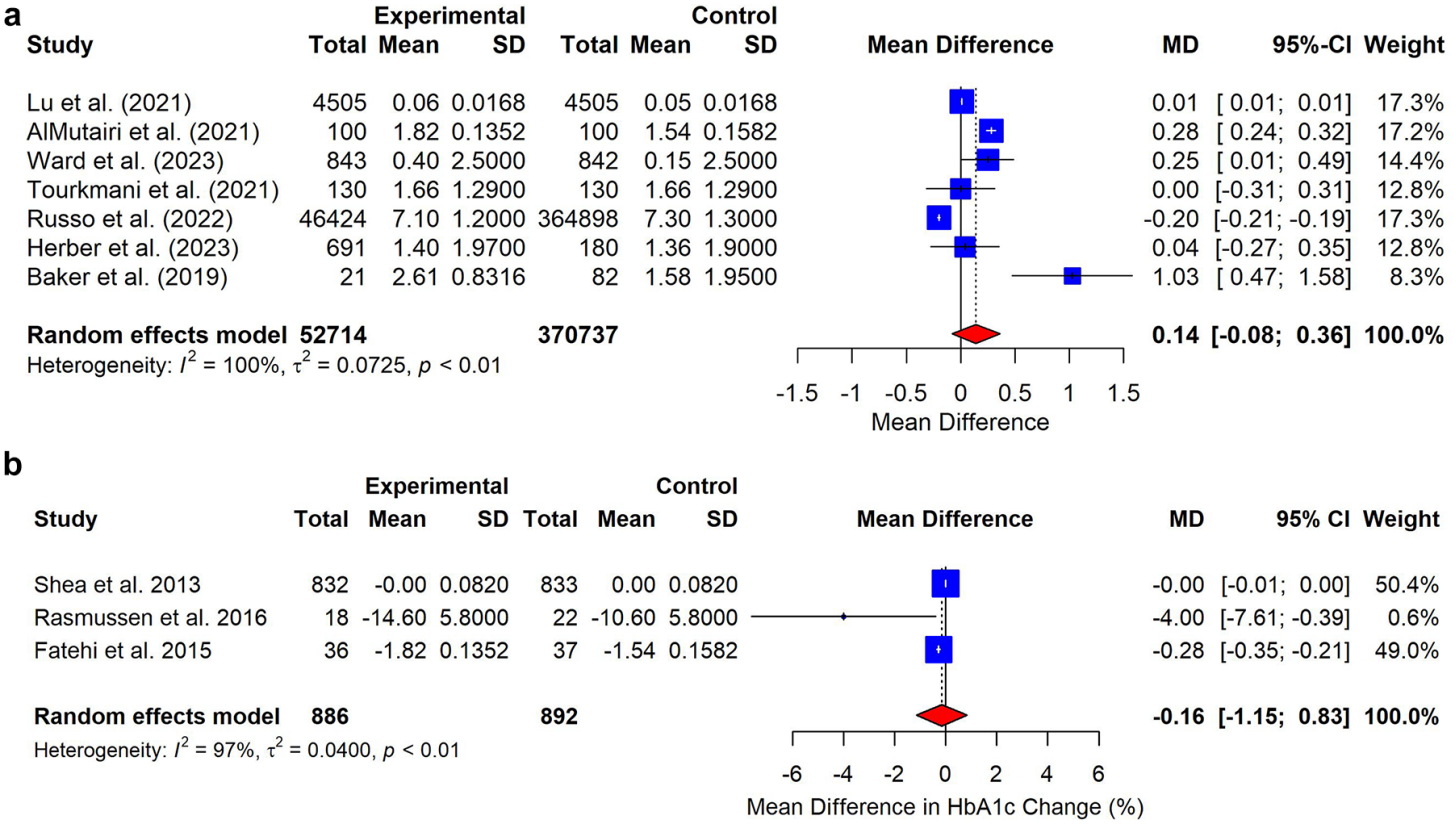
Forest plots of the effect sizes of non-RCTs and RCTs on the impact of virtual consultations versus face-to-face consultations on HbA1c levels. Meta-analysis comparing HbA1c levels in nonrandomized controlled trials (non-RCTs) and randomized controlled trials (RCTs), illustrating the impact of virtual consultations on glycemic control.

Of the 15 studies, most of the studies investigated the clinical outcomes in T2D management.^38-45,47,48,51,52^ Key outcome measures included HbA1c levels,^38-45,47,48,51,52^ blood pressure (BP),^40,47^ and lipid profile.^40,47^ The results demonstrated a range of impacts: BP was examined by two studies,^40,47^ both showing no statistically significant differences between virtual and face-to-face care, in what concerns either systolic BP values (*P* = 0.400 and *P* = 0.430, respectively)^40,47^ or diastolic BP values. Lipid profiles showed no statistically significant differences in total cholesterol change (-1.1±18.6 mg/dL for face-to-face consultations vs -3.7 ± 22.5 for VC, *P* = 0.51) nor triglycerides change (-30.1 ± 38.9 mg/dL for face-to-face consultations vs -14.8 ± 45.7 for VC, *P* = 0.09). Importantly, all studies corroborated there are no statistically significant differences between VC and face-to-face care (Appendix D). The meta-analysis of the eight studies that reported HbA1c, showed no significant difference between VC and face-to-face care in reducing HbA1c levels. The pooled MD in non-RCT studies was 0.1408 (95% CI: -0.0787; 0.3604, *P* = 0.21) indicating a nonsignificant reduction in the HbA1c levels overall. Similarly, the meta-analysis of the RCTs only showed no significant difference, with a pooled SMD of 0.16 (95% CI: -1.15 to 0.83, *P* = 0.55), further corroborating that VC are equally effective to face-to-face care, even when considering only RCTs (Figure 2). The funnel plot did not show significant asymmetry, indicating a minimal bias, with few outliers (Appendix E). Significant heterogeneity was observed among the included studies, with an I^2^ value of 100% for non-RCTs, and 97% for RCTs. The between study-study variance (Tau^2^) was calculated to be 0.04 for non-RCTs and 0.0.62 for RCTs. The sensitivity analysis (a leave-one-out) results were consistent with the main analysis (Appendix F).

Similarly, no significant differences were found in HbA1c levels when performing the meta-analysis including low risk studies only (pooled effect size 0.69; 95% CI: -0.50 to 1.89, *P* = .25), and for the moderate risk studies (pooled effect size 1.07, 95% CI: -0.12 to 2.26, *P* = 0.08) (Appendix G).

### Safety

Out of the 15 studies, three partially assessed the safety of the VC,^49^ with mixed outcomes on patient safety and risk management. In what concerns preventative activities, Lu et al,^40^ reported that the rates of statin and ACE/ARB use and urine microalbumin testing were higher in VC, with an increase in statin prescriptions of 5.1% and a 5.3% increase in ACEi/ARB prescriptions, alongside a 4.6% increase in urine microalbumin testing. One study^50^ identified that while VC was associated with a lower risk of infection in in-person visits, they lacked in providing comprehensive physical examinations, such as for foot ulcers, neuropathy, and some cardiovascular measures (eg, blood pressure monitoring and heart rate variability). While Russo et al’s^43^ study suggests that VC can effectively manage glycemic levels and other metabolic parameters with no significant increase in adverse outcomes or diabetic complications compared with traditional care, it also acknowledges the lack of an in-person physical examination as a safety concern. In another study, the inability to conduct physical examinations virtually was reported as a safety concern by 19% of participants.^43,50^

### Efficiency

Three studies reported improvements in efficiency-related outcomes in the VC group.^39,40,49^ AlMutairi et al found that the total cost of treatment was significantly lower for VC, with an average reduction of $135 (95% CI: -$216 to -$54, *P* < .05). Another study,^39^ found that VC facilitated medication adjustment and provided a more timely alteration of the treatment plan (Table 3).

**Table 3.** Comparative Outcomes of Face-to-Face and Virtual Consultations Across the Included Studies.

Author, year	Outcomes	Face to face	Virtual consultation	Mean difference (95% CI)	
Efficiency					
AlMutairi M et al, 2021	Total treatment cost of USD	$675 ± $81	$540 ± $68	-$135 (-$216 to -$54), *P* < .05*	
Tourkmani et al, 2023	Frequency of in-person visits	1 visit/1-2 weeks	1-2 visits in four months for 64% of patients.	NA	
Beamish et al, 2023	Travel time	Eliminating costs associated with travel and potentially reducing indirect costs such as taking time off work.		NA	
		The total time reduced including the wait times.			
	Total time Spent	42% of patients found waiting times equally well addressed in both modalities.			
		Scheduling Flexibility, more timely appointments			
Safety					
Amy et al, 2021	Prescribed statin, %	68.0 to 69.6	66.7 to 73.3	5.1% (2.4, 7.7)	
	Prescribed ACEi/ARB, %	62.7 to 62.9	58.9 to 64.4	5.3% (2.5, 8.2)	
	Urine microalbumin, %	52.3 to 51.0	48.9 to 52.2	4.6% (1.7, 7.5)	
Timelessness					
Baker et al, 2019	Time to initial visit (days)	Previrtual care	Postvirtual care implementation	60.3 (*P* < .0001*)	
		106.3 ± 24.5	46 ± 35.3		
Beamish et al, 2023	Timeliness
			Reduced wait time		
			Uncertain waiting times		
			Difficulties in scheduling follow-ups.		
**Patient**-centeredness					
Baker et al, 2019	Satisfaction by patient group	<40 years	40-55 years	56-65 years	>65 years:
		High	Moderate	Moderate	Lower
Robinson et al, 2016	Virtual visits overall satisfaction	83% rated as helpful and convenient.			
	Virtual visits’ effectiveness	76% agreed FaceTime improved their care.			
	Technical issues experienced	20% reported connectivity/screen issues.			
	Willingness to continue using virtual	60% would continue poststudy			
Equity					
Baker et al, 2019		<40 years	40-55 years	56-65 years	>65 years
	Mean HbA1c change, %	-1.20%	-1.00%	-0.80%	-0.70%
	Changes in BP and lipids	Significantly improved	Improved	Improved	Slightly improved
Amy et al, 2021	Access to care	Lack to of access to care due to doctor shortage is a significant factor of virtual care in rural areas.			
		Increasing the access to primary care by rural Veterans as V-IMPACT predominantly utilizes videoconferencing			
	Rural vs Urban disparities	Higher rate of mortality from preventable chronic conditions in rural communities such as T2D in comparison to urban			
	Patient demographic	Patients who access virtual care were more likely to be white and living in a rural area.			
	Sustainability of primary care	The virtual care highlights the opportunity for innovative strategies to support the primary care in rural areas			

Tourkmani et al^52^ found that patients under face-to-face care typically required in-person visits every one to two weeks, versus only one or two with VC during the 4-month study period. However, one study highlighted a drawback of VC and reported that 20% of the participants experienced technical disruptions such as connectivity issues, which led to wasted time during consultations.^50^ Table 3 shows the comparative outcomes of Face-to-Face and VC across the included studies.

### Patient Centeredness

Two studies^49,50^ evaluated aspects related to patient-centeredness. Robinson et al^50^ found that 83% (n = 33) of participants rated virtual visits as helpful and convenient, and 76% (n = 30) of the patients felt that using FaceTime for consultations improved their diabetes care by making it more interactive and engaging. However, 20% of the participants experienced technical issues such as connectivity or screen display problems. Despite these issues, a majority (60%) expressed willingness to continue using virtual care post-study (Table 3). Beamish et al,^49^ reported patient perspectives on virtual care, highlighting that most patients (83%) perceived VC as an effective way to manage their diabetes and were either “very satisfied” or “satisfied” with their experience during the COVID-19 pandemic.

### Timeliness

In one of the 15 included studies,^41^ the mean time to initial visit decreased from 106.3 days in the pre-virtual care setup to 46 days postimplementation of VC, reflecting a mean difference of 60.3 days (Table 3). However, Beamish et al^49^ reported mixed experiences regarding the timeliness of VC, while some patients reported a reduction of waiting times and quicker appointments (n = 23, 9%), a few (n = 7, 3%) faced challenges such as uncertainty about delays during appointments, and others (n = 12,5%) reported difficulties in scheduling follow-ups appointments and tests.

### Equity

In a study by Beamish et al,^49^ 4% of the study participants (n = 10), highlighted the benefits of virtual appointments for patients with mobility issues or living in rural areas. However, 14 patients (6%) reported barriers to access, including challenges of telephone consultations for patients with impaired hearing. In the Baker et al^41^ study, younger patients (<40 years) reported higher satisfaction with VC (90% satisfaction), and greater clinical improvement in HbA1c levels, with a reduction of 2.6%. In contrast, older patients (>65 years) reported lower satisfaction (60%) and showed only a modest reduction in HbA1c (0.5% reduction, SD = 0.6).

## Discussion

### Key Findings

Our review highlights the impact of VC on the quality of care for T2D. VCs were comparable with face-to-face care in terms of effectiveness, efficiency, patient-centeredness, and timeliness, demonstrating comparable clinical outcomes, reduced costs, high patient satisfaction, and quicker delivery of care. However, mixed evidence was observed for safety, due to limitations in performing physical examinations, and for equity, as older patients and those with lower digital literacy experienced more challenges compared with younger, more digitally proficient individuals. The meta-analysis showed that VCs are just as effective as face-to-face consultations in controlling HbA1c levels. Several studies highlighted the improved efficiency of VCs, reducing the need for frequent in-person visits and lowering travel and treatment costs. While safety outcomes were generally comparable with face-to-face consultations, VCs provided advantages in certain preventative activities (eg, increased statin prescriptions). However, the inability to conduct physical exams was cited as a limitation, raising potential safety concerns regarding comprehensive patient assessment. VCs demonstrated high levels of patient satisfaction due to their convenience and flexibility, with many patients reporting a preference for VC over traditional consultations, particularly during the COVID-19 pandemic. VCs facilitated faster appointment scheduling and quicker treatment adjustments compared with face-to-face consultations, demonstrating improvements in the timeliness of care delivery. There were mixed findings on equity. Younger patients and those with higher digital literacy benefited more from VCs compared with older adults, who experienced barriers such as lower satisfaction and technical difficulties. The digital divide remains a significant issue, particularly among older adults and individuals from socioeconomically disadvantaged backgrounds. The main findings of the review are presented in Box 1.

**Box 1.** Summary of the Review Main Findings.

**Effectiveness**
VCs are similar to face-to-face care in controlling HbA1c levels and managing T2D, with no significant differences in clinical outcomes.
**Efficiency**
VCs reduce the need for in-person visits, lowering travel times and treatment costs, while improving resource utilization and overall healthcare system efficiency.
**Safety**
Comparable outcomes to Face-to-face; however, concerns exist due to the inability to perform physical exams during VCs, which may affect comprehensive assessments.
**Patient-Centeredness**
High patient satisfaction due to the convenience and flexibility of VCs, with many patients preferring them over traditional consultations, especially during the COVID-19 pandemic.
**Timeliness**
VCs provide quicker communication between patients and providers, enabling faster treatment adjustments and reducing the time to the first consultation.
**Equity**
Mixed evidence: Younger, digitally proficient patients experience better outcomes, while older adults and those with lower digital literacy face barriers such as technical difficulties and lower satisfaction. The digital divide remains a significant issue.

### Clinical Effectiveness

The consistent control of HbA1c levels through VC mirrors the results of prior studies,^18,54,55^ which have also reported equivalent or superior outcomes compared with traditional care. These results suggest that VC can maintain the quality of clinical outcomes in managing T2D and can be as effective as face-to-face interactions. These findings align with those of other systematic reviews,^56-58^ which demonstrated that VC could be as effective as face-to-face care in the context of primary care.

Although one of these reviews focused on primary care in general and was not specific to T2D, its outcomes suggest the integration of VC into routine diabetes care is possible given the similarities in the management needs and chronic nature of T2D.

Another systematic review^20^ focused on synchronous teleconsultation reported similar findings reinforcing the potential of real-time remote consultations in chronic diseases such as T2D. However, other systematic reviews,^58,59^ found mixed results, with some studies reporting improvements while others did not show significant improvement in HbA1c.^60^ This suggests that the effectiveness of VC in glycemic control may vary depending on the specific intervention and patient population.

The findings from the review demonstrate no statistically significant differences between VC and face-to-face care in controlling blood pressure. This is consistent with the results from another review,^61^ which also found that telehealth interventions did not significantly reduce systolic blood pressure (WMD -2.3 mmHg, *P* = 0.527) or diastolic blood pressure (WMD -1.71 mmHg, *P* = 0.163) when compared with usual care. Similarly, a review by Zhang et al^20^ conducted a meta-analysis of VC and found no significant effect on systolic blood pressure, diastolic blood pressure, or body mass index (BMI), aligning with the conclusions of the current review. The impact on lipid profiles was minimal across current and previous studies,^60^ where VC also showed no significant impact on lipid parameters such as total cholesterol and triglycerides compared with usual care. Another review^18^ further support these findings, noting that VC for T2D were effective in maintaining glycemic control but did not emphasize improvements in lipid profiles. Zhang et al,^20^ similarly, reported no significant effect on low-density lipoprotein cholesterol (LDL-C), reinforcing the conclusion that virtual consultations do not provide advantages over traditional care in managing lipid profiles.

### Safety and Quality Considerations

The findings of this review indicated mixed safety outcomes associated with VC. While VC was associated with increased preventative activities, such as higher rates of statin and ACE/ARB prescriptions and urine microalbumin testing, concerns were raised regarding the lack of comprehensive physical examinations, particularly for conditions like foot ulcers, neuropathy, and cardiovascular assessments. This aligns with findings from other studies,^22,59^ which similarly highlighted the limitations of remote care in performing thorough physical exams. Both studies emphasized the potential safety risks of VC without in-person follow-ups, although they noted the benefit of reducing infection exposure, particularly during the COVID-19 pandemic. Other studies also stated these concerns,^61,62^ recognizing that while VC effectively manages glycemic control and reduces infection risk, the inability to perform physical assessments remains a significant limitation. These findings suggest that while VC offers certain safety benefits, a hybrid approach that combines virtual care with periodic in-person evaluations may be necessary to ensure comprehensive patient safety.

### Cost-Effectiveness and Health Care Resources Utilization

Efficiency gains were consistently reported across current and previous studies.^18,20,60,61^ The noted reduction in logistical burdens aligns with previous research that has emphasised the potential for remote consultations to streamline healthcare delivery.^18^ By minimizing the frequency of in-person visits, VC offer more cost-effective solutions, aligned with earlier findings that suggest VC provide resource-efficient health care.^17,19,63,64^ The literature reported similar findings in terms of the cost effectiveness of VC, these findings should influence funding reform decisions for virtual care for T2D and consider a broader impact when planning remote services.^60^ Our findings align with and complement a recent study by MacPherson et al^65^ that examined virtual care interventions for chronic conditions, including diabetes. Both reviews found remote and virtual support to be effective care modalities. MacPherson et al study combined patient utilization profiles with evidence synthesis, identifying populations with the highest acute loads based on admission rates, readmission rates, and length of stay. This approach revealed that diabetes was among the conditions with the highest healthcare utilization, validating our focus on this condition. Their study uniquely combined patient utilization profiles with evidence synthesis to inform implementation priorities, while our review offers a more detailed examination of the clinical effectiveness across the six domains of quality care. Both reviews consistently demonstrate that VC are comparable with face-to-face care in terms of clinical outcomes and patient satisfaction, strengthening the evidence base for virtual care implementation in chronic disease management.

High levels of patient satisfaction with VC have been widely documented,^66,67^ which suggested that VC are a feasible and acceptable option for T2D patients. However, issues with technology usability, the time required to manage virtual sessions, and the need for different communication skills, highlight some of the challenges for both patients and providers.^68^ The high level of patient satisfaction in this review aligned with the findings of other reviews that emphasized the importance of maintaining a supportive infrastructure, including access to devices and reliable connectivity to ensure continued success and patient-centeredness in VC. Adequate infrastructure remains crucial to sustaining a high level of satisfaction and expanding access to remote care. Improvement in patient-caregiver interactions and positive feedback on the ease of use were reported by a few studies. While the current review did not include the perspective of healthcare providers, previous studies reported that general practitioners found remote consultations practical and useful.^69^

In the current review, faster communication and more immediate adjustments to treatment plans were seen, although the survey used in the included study was not formally validated, which might affect the interpretation of these findings. Despite this limitation, the observed benefits support earlier studies suggesting that VC can enhance the timeliness of care.^70,71^ This is particularly valuable in chronic disease management such as for T2D, where real-time and timely interventions between patients and healthcare providers allow immediate feedback and quicker decision making.^20,65,69,72,73^ While digital consultations facilitate quicker access to care by improving the speed and responsiveness of healthcare delivery, they were often hindered by regulatory and infrastructure challenges.^74^

### Digital Divide and Health Care Equity

This review identified significant disparities with VC across different demographic groups, particularly the age-related disparities affecting older adults and those with limited digital literacy, while providing advantages of access to care, especially for individuals with transportation issues or living in rural areas. Our findings align and expand upon others research,^61,75^ emphasizing that VC, once considered innovative, have now become a common avenue of care, particularly among younger and technologically literate populations who showed higher satisfaction and greater clinical improvement in HbA1c levels.^76,77^ The findings of this review align with those reported by the systematic review on barriers and facilitators influencing remote consultations^68^ which highlighted that younger and individuals with higher digital literacy are more likely to engage with and benefit from remote consultations.^78^ While none of the studies definitively conclude that VCs have worsened existing disparities compared with face-to-face, they all highlight the risk of exacerbating these inequities if the challenges faced by older and less digitally literate populations are not adequately addressed.^76,79^

## Strength and Limitations

This systematic review and meta-analysis has several strengths. To our knowledge, this is the first systematic review that maps the six domains of quality of care to investigate the impact of VC on T2D. The inclusion of diverse study designs, such as retrospective cohort studies, randomized controlled trials, and cross-sectional studies, provides a broad perspective on the available evidence. The use of the NAM quality-of-care framework allows for a structured assessment of VC and ensures that all key dimensions of quality care are comprehensively addressed. In addition, the review includes a large sample size of 821 014 participants which strengthens the findings. In addition, A comprehensive search strategy was constructed, likely resulting in the inclusion of the vast majority of relevant studies, thereby enhancing the robustness of the review.

There are several limitations to consider. Significant heterogeneity among the included studies poses challenges for interpreting the pooled results, we conducted a subgroup analysis and sensitivity analysis to explore and report these results. Many studies exhibited a moderate to high risk of bias, which may affect the reliability of the meta-analysis conclusions, the risk of bias was systematically assessed using MMAT (Appendix B), and results were analyzed separately based on bias levels (Appendix G). Safety aspects were not thoroughly explored, particularly regarding potential adverse outcomes. Disparities in digital literacy and access to technology were noted, especially among older adults and individuals from lower socioeconomic backgrounds, potentially limiting the Generalizability of the findings. While this was a limitation of included studies, our review underscores the need for policies aimed at bridging the digital divide, which is also discussed in the implications section. Furthermore, most studies focused on short-term outcomes, with limited information on the long-term impact on T2D management. The emphasis in our recommendations is on longitudinal studies to evaluate the sustained effectiveness and safety of virtual care models. Finally, one study accounted for nearly 90% (733 016/821 014) of the participants in the included studies.

### Implications for Research, Practice, and Policy

Further research is needed to explore the long-term outcomes of VC, particularly their impact on different demographics, safety implications, and the potential for improving equity. The heterogeneity of the current VC suggests that future studies should focus on standardizing remote consultations to better compare outcomes across different settings and populations.

Frameworks and guidelines are needed to support the widespread adoption of VC while ensuring data security and patient privacy. Policies should also aim to bridge the digital divide by providing resources and training to improve digital literacy among older adults and socioeconomically disadvantaged groups. This is particularly important for older adults, who may be less familiar with digital platforms but could greatly benefit from the convenience and accessibility of VC. In addition, telephone consultations are a critical component of VC, particularly for elderly patients with mobility issues, frailty or multimorbidity. By supporting telephone consultations, policies can ensure that these vulnerable populations receive consistent and accessible care.

Research on the common clusters and epidemiology of diabetes highlights the diverse and often younger populations affected by the disease^6,80^ and the increased prevalence of T2D among working-age individuals^81^ necessitates flexible care delivery models. The findings of our review confirmed the uptake of VC by younger patients, suggesting that VC provides a solution by offering flexible appointments and reducing the need for travel. This leads to better engagement and adherence to treatment plans. Policies that promote flexible scheduling for VC can help working-age patients manage their diabetes more effectively while maintaining their professional responsibilities.

## Conclusion

This systematic review and meta-analysis provide evidence that VC yields similar clinical outcomes compared with face-to-face consultations. The review also highlights benefits in terms of efficiency, patient satisfaction, and timeliness of care. However, disparities in digital literacy and access to technology, especially among older adults and socioeconomically disadvantaged groups, present challenges that must be addressed. Policies should focus on improving digital literacy and access to online services, thereby ensuring equitable access to VC to maximize its potential benefits. Further research is needed to explore the long-term impact and safety of VC in T2D management. VC offers a viable alternative to traditional care methods, providing flexible, efficient, and satisfactory care for patients with T2D. Addressing the identified challenges can enhance the integration and effectiveness of VC in the routine care of people with T2D.

## Supplemental Material

sj-docx-1-dst-10.1177_19322968251316585 – Supplemental material for The Impact of Virtual Consultations on Quality of Care for Patients With Type 2 Diabetes: A Systematic Review and Meta-Analysis Supplemental material, sj-docx-1-dst-10.1177_19322968251316585 for The Impact of Virtual Consultations on Quality of Care for Patients With Type 2 Diabetes: A Systematic Review and Meta-Analysis by Reham Aldakhil, Geva Greenfield, Elena Lammila-Escalera, Liliana Laranjo, Benedict W. J. Hayhoe, Azeem Majeed and Ana Luísa Neves in Journal of Diabetes Science and Technology
