# Supplementary material for: The Impact of Virtual Consultations on Quality of Care for Patients With Type 2 Diabetes: A Systematic Review and Meta-Analysis
Source: J Diabetes Sci Technol. 2025 Feb 17;20(4):1435–49. doi: 10.1177/19322968251316585 (PMC11833803; doi:10.1177/19322968251316585)

| *Appendix 1 Search terms and results from databases*  Search 05/12/2024 (Limitation 2010_2024) | | | |
| --- | --- | --- | --- |
| Database | **Concept** | **Search Terms** | **Results** |
| MEDLINE by Ovid | Virtual Consultation | 1. (telemedicine or tele medicine or telehealth or tele health or telecare or tele care or teleconsult* or ((virtual* or remote* or telephon* or phone* or video* or online) adj3 (consult* or appointment*))).mp. [mp=title, abstract, full text, caption text] 2. (telemedicine or "remote consultation").mp. 3. 1 or 2 | 72842 |
|  | Type 2 Diabetes | 1. ("Diabetes Mellitus" or "Type 2 Diabetes Mellitus" or "diabetes type 2" or "T2DM" or "T2D" or "Type two diabetes" or "diabetes Type II" or "Type 2 Diabet*").mp. [mp=title, abstract, full text, caption text] 2. ("Non-Insulin-Dependent Diabetes Mellitus" or "NIDDM" or "non insulin-dependent diabetes" or "NIDD").mp. [mp=title, abstract, full text, caption text] 3. ("Adult-Onset Diabetes" or "Mature-Onset Diabetes").mp. [mp=title, abstract, full text, caption text] 4. 4 or 5 or 6 | 510379 |
|  | **MOI domains:**  Patient-centred | 1. (((experience* or satisfaction) adj4 (patient* or consumer* or client* or survey* or questionnaire*)) or PREM* or "patient-reported experience measure*" or "patient-centred*" or "person-centred*").mp. [mp=title, abstract, full text, caption text] 2. ("Patient Satisfaction" or "Satisfaction of Patients" or "Patient Contentment" or "Patient Experience" or "Patient Perception" or "Quality of Care" or "Healthcare Quality" or "Healthcare Experience").mp. [mp=title, abstract, full text, caption text] 3. 8 or 9 | 255337 |
|  | Effectiveness | 1. (("Treatment Outcome" or "Health" or "Clinical" or "Treatment") adj3 ("Outcome" or "Effective" or "Efficacy")).mp. [mp=title, abstract, full text, caption text] | 464374 |
|  | Safety | 1. ((patient adj3 (safety or harm)) or misdiagnos* or "safety manag*" or (accident* adj2 prevent*) or error* or "medication reconcil*" or "near miss*").mp. [mp=title, abstract, full text, caption text] 2. (patient harm or patient safety or "Diagnostic Errors").mp. [mp=title, abstract, full text, caption text] 3. 12 or 13 | 365,084 |
|  | Efficiency | 1. (efficiency or economic* or cost* or expenditure* or charge* or (number adj3 appointment*) or (number adj3 admission*) or (number adj3 consultation*)).mp. [mp=title, abstract, full text, caption text] | 802084 |
|  | Timeliness | 1. (wait* list* or wait* time* or timeliness).mp. [mp=title, abstract, full text, caption text] 2. ("Time-to-Treatment" or "Waiting Lists").mp. [mp=title, abstract, full text, caption text] 3. 16 or 17 | 756,358 |
|  | Equity | 1. ((health* or health care or access) adj3 (equity or disparit* or inequit* or inequalit* or equality or gap)).mp. [mp=title, abstract, full text, caption text] 2. "Health Equity".mp. [mp=title, abstract, full text, caption text] 3. 19 or 20 | 279,439 |
|  |  | 1. 10 or 11 or 14 or 15 or 18 or 21 2. 3 and 7 and 22 |  |
|  |  | 1. limit 23 to yr="2010 -Current" | 941 |

| Search date 06/12/2024 (Limitation 2010_2024) | | | |
| --- | --- | --- | --- |
| Database | **Concept** | **Search Terms** | **Results** |
| Cochrane | Virtual Consultation | 1. (telemedicine OR "tele medicine" OR telehealth OR "tele health" OR telecare OR "tele care" OR teleconsult* OR ((virtual* OR remote* OR telephon* OR phone* OR video* OR online ) NEAR/3 (consult* OR appointment* ))) 2. "telemedicine" OR "remote consultation" 3. #1 OR #2 | 11240 |
|  | Type 2 Diabetes | 1. ("Diabetes Mellitus" OR "Type 2 Diabetes Mellitus" OR "diabetes type 2" OR T2DM OR T2D OR "Type two diabetes" OR "diabetes Type II" OR "Type 2 diabetes" OR "Type 2" AND "diabetes") 2. ("Non-Insulin-Dependent Diabetes Mellitus" OR NIDDM OR "non insulin-dependent diabetes" OR NIDD ) 3. ("Adult-Onset Diabetes" OR "Mature-Onset Diabetes" ) 4. #4 OR #5 OR #6 | 33405 |
|  | **MOI domains:**  Patient-centred | 1. (((experience* OR satisfaction ) NEAR/4 (patient* OR consumer* OR client* OR survey* OR questionnaire* )) OR PREM* OR ("patient-reported experience" NEXT measure*) OR patient-centred* OR person-centred* ) 2. [mh ^"Patient Satisfaction"] 3. #8 OR #9 | 66265 |
|  | Effectiveness | 11. "Treatment Outcome" OR (health OR clinical* OR treatment*) AND (outcome* OR effective* OR efficacy) | 449413 |
|  | Safety | 12. (patient AND (safety OR harm)) OR misdiagnos* OR (safety AND manag*) OR (accident* AND prevent*) OR error* OR (medication AND reconcil*) OR (near AND miss*)  13.[mh ^"patient harm"] OR [mh ^"patient safety"] OR [mh ^"Diagnostic Errors"]  14.#12 OR #13 | 5060 |
|  | Efficiency | 15.(efficiency OR economic* OR cost* OR expenditure* OR charge* OR (number NEAR/3 appointment* ) OR (number NEAR/3 admission* ) OR (number NEAR/3 consultation* )) | 56937 |
|  | Timeliness | 16. "waiting list" OR "wait time" OR "waiting time" OR timeliness):ti,ab,kw  17. ("Time to Treatment" OR "Waiting Lists"):ti,ab,kw  18.#16 OR #17 | 6434 |
|  | Equity | 19. ((health* OR "health care" OR access) AND (equity OR disparit* OR inequit* OR inequalit* OR equality OR gap)):ti,ab,kw  20. ("Health Equity"):ti,ab,kw  21.#19 OR #20 | 15415 |
|  |  | 22.#10 OR #11 OR #14 OR #15 OR #18 OR #21 | 529732 |
|  |  | 23.#3 AND #7 AND #22 | 786 |

| Search date 05/12/2024 Limitation 2010_2024 | | | | | |
| --- | --- | --- | --- | --- | --- |
| Database | Concept | Search Terms | | Results | |
| CINAHL via EBSCO | Virtual Consultation | 1. (telemedicine OR "tele medicine" OR telehealth OR "tele health" OR telecare OR "tele care" OR teleconsult* OR (virtual* AND (consult* OR appointment*))) 2. (TX telemedicine OR TX "remote consultation") 3. 1 or 2 | | 19535 | |
|  | Type 2 Diabetes | 1. ("Diabetes Mellitus" or "Type 2 Diabetes Mellitus" or "diabetes type 2" or "T2DM" or "T2D" or "Type two diabetes" or "diabetes Type II" or "Type 2 Diabet*").mp. [mp=title, abstract, full text, caption text] 2. ("Non-Insulin-Dependent Diabetes Mellitus" OR NIDDM OR "non insulin-dependent diabetes" OR NIDD) 3. ("Adult-Onset Diabetes" OR "Mature-Onset Diabetes") 4. 4 or 5 or 6 | | 56638 | |
|  | **MOI domains:**  Patient-centred | 1. (experience* OR satisfaction) AND (patient* OR consumer* OR client* OR survey* OR questionnaire*) OR "patient-reported experience measures" OR patient-centred OR person-centred 2. ("Patient Satisfaction" OR "Satisfaction of Patients" OR "Patient Contentment" OR "Patient Experience" OR "Patient Perception" OR "Quality of Care" OR "Healthcare Quality" OR "Healthcare Experience") AND (TI AB). 3. 8 or 9 | | 170940 | |
|  | Effectiveness | 1. "Treatment Outcome" OR (health OR clinical* OR treatment*) AND (outcome* OR effective* OR efficacy) | | 498155 | |
|  | Safety | 1. (patient AND (safety OR harm)) OR misdiagnos* OR (safety AND management) OR (accident* AND prevention) OR error* OR (medication AND reconciliation) OR "near miss" 2. (TI "patient harm" OR AB "patient harm" OR MH "Patient Harm") OR   (TI "patient safety" OR AB "patient safety" OR MH "Patient Safety") OR  (TI "Diagnostic Errors" OR AB "Diagnostic Errors" OR MH "Diagnostic Errors")   1. 12 or 13 | | 118588 | |
|  | Efficiency | 1. (efficiency OR economic* OR cost* OR expenditure* OR charge* OR (number N3 appointment*) OR (number N3 admission*) OR (number N3 consultation*)) | | 154215 | |
|  | Timeliness | 1. ("waiting list*" OR "waiting time*" OR timeliness) AND (TI AB) 2. ("Time-to-Treatment" OR "Waiting Lists") AND (TI AB TX) 3. 16 or 17 | | 7316 | |
|  | Equity | 1. (health* OR "health care" OR access) AND (equity OR disparity* OR inequity* OR inequality* OR equality OR gap) 2. "Health Equity" AND (TI "Health Equity" OR AB "Health Equity") 3. 19 or 20 | | 38009 | |
|  |  | 1. 10 or 11 or 14 or 15 or 18 or 21 | | 1,063,396 | |
|  |  | 1. 3 and 7 and 22 | | 540 | |
|  |  |  |  |  |  |
| Search date 05/12/2024 Limitation 2010_2024 | | | | | |
| Database | Concept | Search Terms | Results | |  |
| Web of Science | Virtual Consultation | 1. (telemedicine OR "tele medicine" OR telehealth OR "tele health" OR telecare OR "tele care" OR teleconsult* OR (virtual* OR remote* OR telephon* OR phone* OR video* OR online )) 2. (consult* OR appointment*) 3. 1 or 2 |  | |  |
|  | Type 2 Diabetes | 1. ("Diabetes Mellitus" OR "Type 2 Diabetes Mellitus" OR "diabetes type 2" OR T2DM OR T2D OR "Type two diabetes" OR "diabetes Type II" OR diabet* OR "Type 2 Diabet*" OR T2DM OR T2D) 2. ("Non-Insulin-Dependent Diabetes Mellitus" OR NIDDM OR "non insulin-dependent diabetes" OR NIDD) 3. ("Adult-Onset Diabetes" OR "Mature-Onset Diabetes") 4. 4 or 5 or 6 | 179238 | |  |
|  | **MOI domains:**  Patient-centred | 1. ((experience* OR satisfaction ) AND (patient* OR consumer* OR client* OR survey* OR questionnaire* )) OR PREM* OR "patient-reported experience measure*" OR patient-centred* OR person-centred* 2. ("Patient Satisfaction" OR "Satisfaction of Patients" OR "Patient Contentment" OR "Patient Experience" OR "Patient Perception" OR "Quality of Care" OR "Healthcare Quality" OR "Healthcare Experience") 3. 8 OR 9 | 414642 | |  |
|  | Effectiveness | 1. (health OR clinical* OR treatment* ) AND (outcome* OR effective* OR efficacy) | 953686 | |  |
|  | Safety | 1. ((patient AND (safety OR harm )) OR misdiagnos* OR "safety manag*" OR (accident* AND prevent* ) OR error* OR "medication reconcil*" OR "near miss*" 2. ("patient harm" OR "patient safety" OR "Diagnostic Errors") 3. 12 OR 13 | 486752 | |  |
|  | Efficiency | 1. (efficiency OR economic* OR cost* OR expenditure* OR charge* OR (number N3 appointment*) OR (number N3 admission*) OR (number N3 consultation*)) | 1768462 | |  |
|  | Timeliness | 1. ("wait* list*" OR "wait* time*" OR timeliness) 2. ("Time-to-Treatment" OR "Waiting Lists") 3. 16 or 17 | 19391 | |  |
|  | Equity | 1. ((health* OR "health care" OR access ) AND (equity OR disparit* OR inequit* OR inequalit* OR equality OR gap )) 2. "Health Equity" 3. 19 or 20 | 93460 | |  |
|  |  | 1. 10 or 11 or 14 or 15 or 18 or 21 2. 3 and 7 and 22 | 3,898,506  307 | |  |

| Database | Concept | Search Terms | Results |
| --- | --- | --- | --- |
| EMBASE | Virtual Consultation | 1. (telemedicine or tele medicine or telehealth or tele health or telecare or tele care or teleconsult* or ((virtual* or remote* or telephon* or phone* or video* or online) adj3 (consult* or appointment*))).mp 2. (telemedicine or telemedicine or (remote consultation or 'remote consultation')).mp. 3. 1 OR 2 | 104508 |
|  | Type 2 Diabetes | 1. ("diabetes mellitus" or "diabetes mellitus type 2" or "diabetes type 2" or T2DM or T2D or "type two diabetes" or "diabetes type II" or diabet* or ("type 2" adj diabet*)).mp. 2. ("non-insulin-dependent diabetes mellitus" OR NIDDM OR "non insulin-dependent diabetes" OR NIDD OR "ketosis resistant diabetes mellitus").mp. 3. ("adult-onset diabetes" OR "mature-onset diabetes" OR "maturity onset diabetes").mp. 4. 4 OR 5 OR 6 | 1645771 |
|  | **MOI domains:**  Patient-centred | 1. (((experience* OR satisfaction) NEAR/4 (patient* OR consumer* OR client* OR survey* OR questionnaire*)) OR PREM* OR ("patient-reported experience" NEXT measure*) OR patient-centred* OR person-centred*).mp. 2. 'patient satisfaction'/exp 3. 8 OR 9 | 1272899 |
|  | Effectiveness | 1. 'treatment outcome'/exp OR ((health OR clinical* OR treatment*) NEAR/3 (outcome* OR effective* OR efficacy)).mp. | 272113 |
|  | Safety | 1. ((patient NEAR/3 (safety OR harm)) OR misdiagnos* OR ("safety" NEXT manag*) OR (accident* NEAR/2 prevent*) OR error* OR ("medication" NEXT reconcil*) OR ("near" NEXT miss*)).mp. 2. 'patient harm'/exp OR 'patient safety'/exp OR 'diagnostic error'/exp 3. 12 OR 13 | 1119072 |
|  | Efficiency | 1. (efficiency OR economic* OR cost* OR expenditure* OR charge* OR (number NEAR/3 appointment*) OR (number NEAR/3 admission*) OR (number NEAR/3 consultation*)).mp. | 3132235 |
|  | Timeliness | 1. ((wait* NEXT list*) OR (wait* NEXT time*) OR timeliness).mp. 2. 'time to treatment'/exp OR 'waiting list'/exp 3. 16 OR 17 | 157110 |
|  | Equity | 1. ((health* OR "health care" OR access) NEAR/3 (equity OR disparit* OR inequit* OR inequalit* OR equality OR gap)).mp. 2. 'health equity'/exp 3. 19 OR 20 | 161166 |
|  |  | 1. 10 OR 11 OR 14 OR 15 OR 18 OR 21 2. 3 AND 7 AND 22 | 6340561 |
|  |  | Limit: 2010_current | 1104 |

**Search 06/12/2024 Limitation 2010_2024**

Appendix 2 Mixed Methods Risk of Bias Assessment Tool

| **SCREENING QUESTIONS** | | | | | | | | | | | **2. RANDOMIZED CONTROLLED TRIALS** | | | | | | | | | | | | | | | | | | | | | | | **3. NON-RANDOMIZED STUDIES** | | | | | | | | | | | | | | | | | | | | | | | | **4. QUANTITATIVE DESCRIPTIVE STUDIES** | | | | | | | | | | | | | | | | | | | | | | | | **5. MIXED METHODS STUDIES** | | | | | | | | | | | | | | | | | | | | | | | | | | | | | | |  | | | | | | | |  |  |
| --- | --- | --- | --- | --- | --- | --- | --- | --- | --- | --- | --- | --- | --- | --- | --- | --- | --- | --- | --- | --- | --- | --- | --- | --- | --- | --- | --- | --- | --- | --- | --- | --- | --- | --- | --- | --- | --- | --- | --- | --- | --- | --- | --- | --- | --- | --- | --- | --- | --- | --- | --- | --- | --- | --- | --- | --- | --- | --- | --- | --- | --- | --- | --- | --- | --- | --- | --- | --- | --- | --- | --- | --- | --- | --- | --- | --- | --- | --- | --- | --- | --- | --- | --- | --- | --- | --- | --- | --- | --- | --- | --- | --- | --- | --- | --- | --- | --- | --- | --- | --- | --- | --- | --- | --- | --- | --- | --- | --- | --- | --- | --- | --- | --- | --- | --- | --- | --- | --- | --- | --- | --- | --- |
| **Author, Year** | | | S1. Are there clear research questions? | | | | | S2. Do the collected data allow to address the research questions? | | 2.1. Is randomization appropriately performed? | | | | | | 2.2. Are the groups comparable at baseline? | | | | | | 2.3. Are there complete outcome data? | | | | | 2.4. Are outcome assessors blinded to the intervention provided? | | | | 2.5 Did the participants adhere to the assigned intervention? | | | 3.1. Are the participants representative of the target population? | | | | | 3.2. Are measurements appropriate regarding both the outcome and intervention (or exposure)? | | | | 3.3. Are there complete outcome data? | | | | 3.4. Are the confounders accounted for in the design and analysis? | | | | | 3.5. During the study period, is the intervention administered (or exposure occurred) as intended? | | | | | | 4.1. Is the sampling strategy relevant to address the research question? | | | | 4.2. Is the sample representative of the target population? | | | | | | | 4.3. Are the measurements appropriate? | | | | 4.4. Is the risk of nonresponse bias low? | | | | 4.5. Is the statistical analysis appropriate to answer the research question? | | | | | 5.1. Is there an adequate rationale for using a mixed methods design to address the research question? | | | | | | 5.2. Are the different components of the study effectively integrated to answer the research question? | | | | | | 5.3. Are the outputs of the integration of qualitative and quantitative components adequately interpreted? | | | | 5.4. Are divergences and inconsistencies between quantitative and qualitative results adequately addressed? | | | | | | | | 5.5. Do the different components of the study adhere to the quality criteria of each tradition of the methods involved? | | | | | | | **Overall Risk** | | | | | | | | | |
| Adhikari S.; 2023 | | Yes | | | | Yes | | | | |  | | | |  | | | |  | | | | | |  | | | | |  | | | | Yes | | | | | Yes | | Can't tell | | | | | Yes | | | | | Can't tell | | | | | |  | | | |  | | | |  | | |  | | | | | | |  | | | | | | | |  | |  | | | | | | | | | | |  | | |  | | | |  | | | | | | | | | | | Moderate | | | | |  |  |  |  |
| Amy D.; 2021 | | Yes | | | | Yes | | | | |  | | | |  | | | |  | | | | | |  | | | | |  | | | | Yes | | | | | Yes | | Can't tell | | | | | Yes | | | | | Yes | | | | | |  | | | |  | | | |  | | |  | | | | | | |  | | | | | | | |  | |  | | | | | | | | | | |  | | |  | | | |  | | | | | | | | | | | Low | | | | |  |  |  |  |
| AlMutairi M; 2021 | | Yes | | | | Yes | | | | |  | | | |  | | | |  | | | | | |  | | | | |  | | | | Yes | | | | | Yes | | Can't tell | | | | | Yes | | | | | Yes | | | | | |  | | | |  | | | |  | | |  | | | | | | |  | | | | | | | |  | |  | | | | | | | | | | |  | | |  | | | |  | | | | | | | | | | | Low | | | | |  |  |  |  |
| Baker, J. W.; 2019 | | Yes | | | | Yes | | | | |  | | | |  | | | |  | | | | | |  | | | | |  | | | |  | | | | |  | |  | | | | |  | | | | |  | | | | | | Yes | | | | Yes | | | | Yes | | | Can't tell | | | | | | | Yes | | | | | | | |  | |  | | | | | | | | | | |  | | |  | | | |  | | | | | | | | | | | Low | | | | |  |  |  |  |
| Beamish P.; 2023 | | Yes | | | | Yes | | | | |  | | | |  | | | |  | | | | | |  | | | | |  | | | |  | | | | |  | |  | | | | |  | | | | |  | | | | | |  | | | |  | | | |  | | |  | | | | | | |  | | | | | | | | Yes | | Yes | | | | | | | | | | | Yes | | | Can't tell | | | | Yes | | | | | | | | | | | Low | | | | |  |  |  |  |
| Fatehi F.; 2015 | | | Yes | | | Yes | | | | | Yes | | | | | Yes | | | | | | | Can't tell | | | | | No | | | | Yes | | | | |  | | | |  | | | | | |  | | | | |  | | | | | |  | | | | |  | | |  | | | | |  | | | | | |  | |  | | | | | | |  | | | |  | | | | | | | | | |  | | | |  | | | | |  | | | | | Moderate | | |  |  |  |  |  |  |
| Herber E.; 2023 | | | Yes | | | Yes | | | | |  | | | | |  | | | | | | |  | | | | |  | | | |  | | | Can't tell | | | | Yes | | | | Can't tell | | | | | | Yes | | | | | | Can't tell | | | | | | | |  | | |  | | | | |  | | | | | |  | |  | | | | | | |  | | | |  | | | | | | | | | |  | | | |  | | | | |  | | | | | Moderate | | |  |  |  |  |  |  |
| Koh ZWJ; 2023 | | | Yes | | | Yes | | | | |  | | | | |  | | | | | | |  | | | | |  | | | |  | | | Can't tell | | | | Yes | | | | Can't tell | | | | | | Yes | | | | | | Yes | | | | | | | | | |  | | | |  | | | |  | | | | |  | |  | | | | |  | | | | | |  | | | | |  | | | | | | | | | |  | | | |  | | | | Moderate | | |  |  |  |  |  |  |
| Rasmussen O. W.; 2015 | | | Yes | | | Yes | | | | | Yes | | | | | Yes | | | | | | | Yes | | | | | Can't tell | | | | Yes | | | | |  | | | |  | | | | | | |  | | | | |  | | | | | |  | | | | | |  | | | |  | | | |  | | | | |  | |  | | | | |  | | | | | |  | | | | |  | | | | | | | | | |  | | | |  | | | | Low | | |  |  |  |  |  |  |
| Robinson, M. D.; 2016 | | | Yes | | | Yes | | | | |  | | | | |  | | | | | | | | |  | | |  | | | |  | |  | | | | | | |  | | | | | | |  | | | | |  | | | | | |  | | | | | |  | | | |  | | | |  | | | | |  | |  | | | Yes | | | | | Yes | | | | Can't tell | | | | | | | | | Can't tell | | | | | | Can't tell | | | | | | | | Moderate | | | |  |  |  |  |
| Russo, G. T.; 2022 | | | Yes | | | Yes | | | | |  | | | | | | | | |  | | | | |  | | |  | | | |  | | Can't tell | | | | | | | Yes | | | | | | | Can't tell | | | | | Can't tell | | | | | | Can't tell | | | | | |  | | | |  | | | |  | | | | |  | |  | | | | |  | | | | | |  | | | | |  | | | | | | |  | | | | | | |  | | | | | High | | | | | | | |
| Shao, Y. X., 2023 | | | | Yes | | | | Yes | | | | |  | | | | | |  | | | | | | | |  | | | |  | | | | | Yes | | | | | | | | Yes | | | | | | | | | | Can't tell | | | | | | | | Yes | | | | | | | | Yes | | | | |  | | | | | | | | | | | | | |  | | | | |  | | | | |  | | | | | | | | |  | | |  | | | | | Low | | | | |  |  |
| Shea S.; 2013 | | | | Yes | | | | Yes | | | | Yes | | | | | Yes | | | | | Can't tell Yes | | | | | | | | | Yes | | | | |  | | | | | | | |  | | | | | | | | | |  | | | | | | | |  | | | | | | | |  | | | | |  | | | | | | | | | | | | | |  | | | | |  | | | | |  | | | | | | | | |  | | |  | | | | | Low | | | | |  |  |
| Tourkmani, A. M.; 2023 | | | | Yes | | | | Yes | | | | |  | | | | | |  | | | | | | | |  | | | |  | | | | | Yes | | | | | | | | Yes | | | | | | | | | | Can't tell | | | | | | | | Can't tell | | | | | | | | Yes | | | | |  | | | | | | | | | | | | | |  | | | | |  | | | | |  | | | | | | | | |  | | |  | | | | | Moderate | | | | |  |  |
| Ward, L. A.; 2023 | | | | Yes | | | | Yes | | | | |  | | | | | |  | | | | | | | |  | | | |  | | | | | Yes | | | | | | | | Yes | | | | | | | | | | Can't tell | | | | | | | | Can't tell | | | | | | | | Yes | | | | |  | | | | | | | | | | | | | |  | | | | |  | | | | |  | | | | | | | | |  | | |  | | | | | Moderate | | | | | | |

Appendix 3 Risk of bias assessments for non-randomized studies, randomized controlled trials, quantitative studies, and mixed methods studies. The color-coding scheme is as follows: Green for "Low Risk," Yellow for "Moderate Risk," and Red for "High Risk."


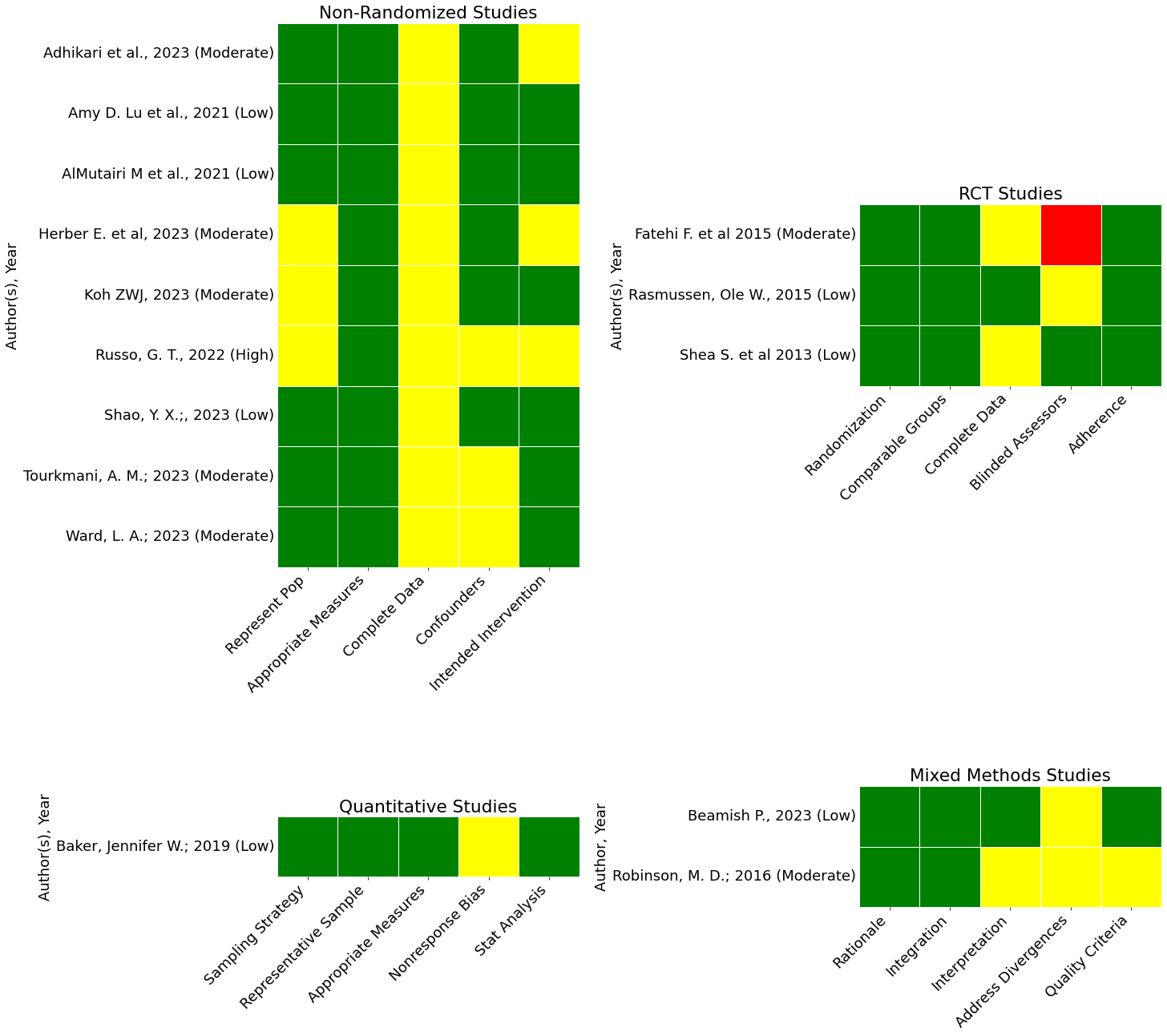


Appendix 4 The effectiveness of face-to-face Vs Virtual consultations

| **Author, year** | **Study design** | **Outcomes** | | **Face to Face** | **Virtual**  **Consultation** | | | **Mean Difference (95% CI)** | |  |
| --- | --- | --- | --- | --- | --- | --- | --- | --- | --- | --- |
| Amy et al, 2021 | Retrospective Cohort Study | HbA1C, mean % | 7.36 to 7.31 | | | 7.33 to 7.27 | −0.008% (−0.055, 0.039) | |  |  |
|  |  | Controlled BP < 140/90, % | 64.7 to 66.8 | | | 65.3 to 67.7 | 0.2% (−1.1, 1.5) | |  |  |
|  |  | Controlled BP < 130/80, % | 31.8 to 34.3 | | | 32.4 to 35.1 | 0.1% (−1.1, 1.4) | |  |  |
|  |  | Prescribed statin, % | 68.0 to 69.6 | | | 66.7 to 73.3 | 5.1% (2.4, 7.7) | |  |  |
|  |  | Prescribed ACEi/ARB, % | 62.7 to 62.9 | | | 58.9 to 64.4 | 5.3% (2.5, 8.2) | |  |  |
|  |  | Urine microalbumin testing, % | 52.3 to 51.0 | | | 48.9 to 52.2 | 4.6% (1.7, 7.5) | |  |  |
| Herber et al, 2023 | Retrospective Cohort Study | HbA1C, mean % | 1.36 ± 1.91 | | | 1.40 ± 1.97 | 0.04 (−0.28, 0.36) | |  |  |
| Baker et al, 2019 | Retrospective Cohort Study | Mean HbA1C reduction  at 3 months | 1.468 ± 1.75 | | | 2.262 ± 2.8 | 0.794 (no CI provided), p = 0.0369* | |  |  |
|  |  | Mean HbA1C reduction  at 6 months | 1.584 ± 1.95 | | | 2.610 ± 3.8 | 1.026 (no CI provided), p = 0.1014 | |  |  |
| AlMutairi et al, 2021 | Retrospective Cohort Study | HbA1C, mean % | 1.54 ± 1.56 | | | 1.82 ± 1.35 | 0.28 (−0.194 to 0.546), p < 0.001* | |  |  |
| Rasmussen et al, 2015 | RCT | HbA1C, mean % | Decrease by 10.6% | | | Decrease by 14.6% | 4.0% (no CI provided), p = 0.016 | |  |  |
|  |  | Mean Blood Glucose reduction, % | Decrease by 13.1 | | | Decreased by 17.6% | 4.5% (no CI provided), p = 0.015 | |  |  |
|  |  | Cholesterol reduction, % |  | | | Decrease by 7.1% | 1.0% (no CI provided), p = 0.044 | |  |  |
|  |  | Systolic BP (mm Hg) | 0 change | | | 0 change | no change (no CI provided), p = 0.4 | |  |  |
|  |  | Diastolic BP (mm Hg) | Decrease by 7 | | | Decrease by 1.2 | 5.8 increase (no CI provided), p = 0.43 | |  |  |
| Shao et al, 2023 | Quasi-experimental | HbA1C, mean % | 7.112 to 7.215 | | | 7.215 to 7.069 | -0.146 (−0.178, −0.114), p < 0.001 | |  |  |
| Ward et al, 2023 | Retrospective Cohort Study | HbA1c % change | 8.00% to 7.85% (−0.15%) | | | 8.16% to 7.76% (−0.40%) | −0.25% (−0.32% to −0.18%), p < 0.001 | |  |  |
| Beamish et al, 2023 | Cross-sectional | HbA1C, mean % | -0.15 (SD = 1.25) | | | -0.21 (SD = 1.4) | p = 0.44 | |  |  |
|  |  | Total Cholesterol change (mg/dL) | -1.1 (SD = 18.6) | | | -3.7 (SD = 22.5) | p = 0.51 | |  |  |
|  |  | Triglycerides change (mg/dL) | -30.1 (SD = 38.9) | | | -14.8 (SD = 45.7) | p = 0.09 | |  |  |
| Abbreviations: HbA1c: Hemoglobin A1c, BP: Blood Pressure, ACEi: Angiotensin-Converting Enzyme Inhibitor,  RCT: Randomised Controlled Study, ARB: Angiotensin II Receptor Blocker, CI: Confidence Interval, SD: Standard Deviation, mg/dL: Milligrams per Decilitre | | | | | | | | | | |

Appendix 5 Funnel Plot for Publication Bias in HbA1c Changes

**
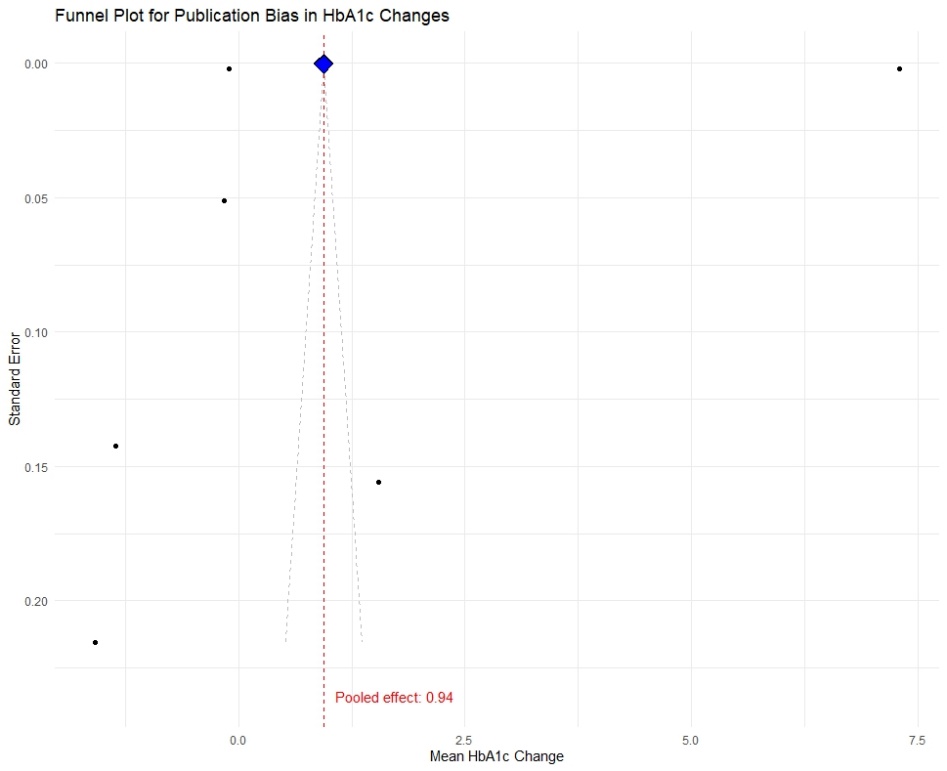
**

Appendix 6 Leave-one-out sensitivity analysis plots showing influence diagnostics for rstudent, dffits, cook's distance (cook.d), covariance ratio (cov.r), tau-squared deletion (tau2.del), QE deletion (QE.del), leverage (hat), and weights (weight).


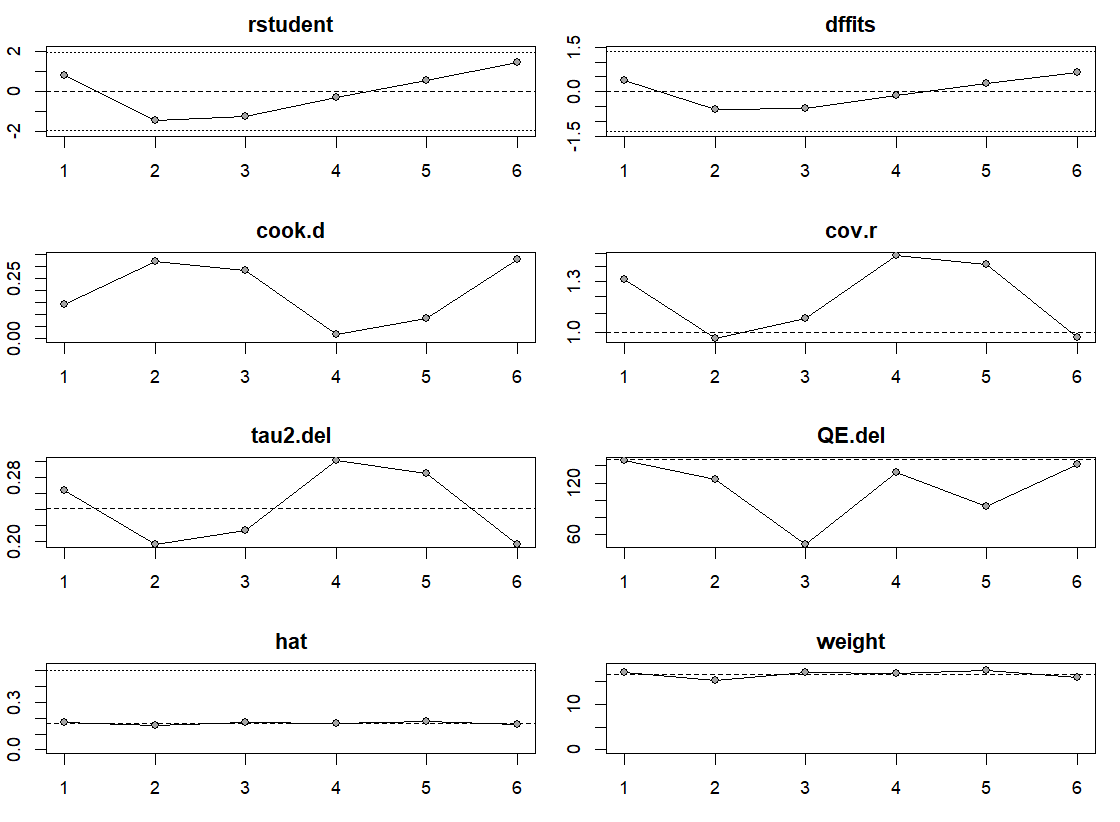


Appendix 7 Forest plots of the effect sizes of different risk categories on the impact of virtual consultations versus face-to-face consultations on HbA1c levels. High-risk studies were excluded as only one study was classified as high risk.


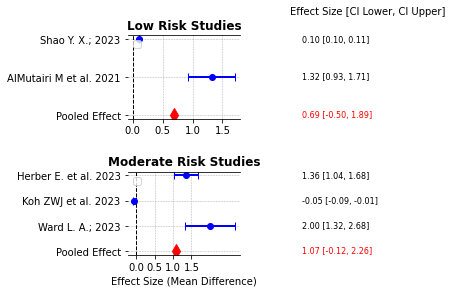

Supplement: sj-docx-1-dst-10.1177_19322968251316585 – Supplemental material for The Impact of Virtual Consultations on Quality of Care for Patients With Type 2 Diabetes: A Systematic Review and Meta-Analysis [file sj-docx-1-dst-10.1177_19322968251316585.docx]
